# Fetal Hydronephrosis: A Narrative Review of Diagnostic Pathways, Prognostic Markers, and Management

**DOI:** 10.7759/cureus.114189

**Published:** 2026-08-09

**Authors:** Ileana V Baltogianni, Melina E Baltogianni, Fotini Fili, Elsa Faviou, Nikolaos Baltogiannis

**Affiliations:** 1 Department of Anesthesiology, General Hospital of Athens “Elpis”, Athens, GRC; 2 Department of Obstetrics and Gynecology, General Hospital “Agios Andreas”, Patra, GRC; 3 Department of Pediatric Surgery, Private Children’s Hospital of “Athens Medical Group”, Athens, GRC; 4 Department of Clinical Biochemistry, Private Diagnostic and Research Center "Protipo MedLabs", Athens, GRC

**Keywords:** congenital urinary anomalies, fetal hydronephrosis, kidney disease, lower urinary tract obstruction, pediatric mri, postnatal evaluation, prenatal ultrasonography, prenatal ultrasound, urinary tract dilation, utd classification

## Abstract

Fetal hydronephrosis is the most frequently detected prenatal urinary tract abnormality and encompasses a broad clinical spectrum, ranging from transient physiological dilation to clinically significant obstructive uropathy with potential long-term renal consequences. The broader term urinary tract dilation integrates renal pelvic and calyceal dilation with abnormalities of the renal parenchyma, ureters, bladder, and amniotic fluid volume, thereby supporting more comprehensive risk stratification. This narrative review synthesizes evidence on fetal urinary tract development, prenatal diagnosis, contemporary classification systems, differential diagnosis, prenatal intervention, postnatal evaluation, management, and long-term outcomes. Particular emphasis is placed on the transition from isolated renal pelvic measurements to multidimensional assessment using the Urinary Tract Dilation classification system, serial imaging, parenchymal findings, and functional evaluation. Most infants with isolated low-risk dilation experience spontaneous improvement or resolution and can be managed conservatively, whereas severe, bilateral, progressive, or complex abnormalities require structured prenatal and postnatal surveillance, selective use of voiding cystourethrography and radionuclide renography, and, in some cases, surgical intervention. Fetal magnetic resonance imaging and urinary or amniotic fluid biomarkers may provide additional information in selected cases; however, biomarkers remain investigational and should not independently guide clinical decisions. Current evidence supports an individualized, risk-based approach that balances early identification of clinically significant obstruction with avoidance of unnecessary investigations and treatment in self-limiting cases.

## Introduction and background

Fetal hydronephrosis (FH) is the most frequently identified urinary tract abnormality during routine prenatal ultrasonographic screening, with reported incidence rates ranging from 0.6% to 4.5% of pregnancies, depending on gestational age at assessment, diagnostic thresholds, and study methodology [1-3]. Advances in prenatal imaging have enabled detection as early as the late first trimester, although most cases are identified during second- and third-trimester evaluations [4]. Bilateral involvement occurs in approximately 20-40% of affected fetuses [1,5], and a consistent male predominance has been reported, with a male-to-female ratio approaching 2:1, reflecting sex-related differences in urinary tract development and susceptibility to obstructive pathology [6].

The term urinary tract dilation (UTD) has been introduced to encompass a broader spectrum of prenatal urinary abnormalities, including hydronephrosis and associated anomalies affecting the renal collecting system, ureters, and bladder. In this context, FH refers primarily to dilation of the renal pelvis and collecting system, whereas UTD is a broader standardized term that also incorporates calyceal dilation, renal parenchymal abnormalities, ureteral dilation, bladder findings, and amniotic fluid volume [5]. Population-based studies have shown that UTD is the most common prenatal ultrasonographic finding overall, affecting approximately 1%-2% of pregnancies and accounting for nearly 10% of congenital structural anomalies detected in utero [7]. Although most cases - estimated at 70%-80% - represent transient or physiological dilatation that resolves spontaneously during fetal life or early infancy without adverse long-term consequences [7], a clinically relevant subset is associated with congenital obstructive uropathies, vesicoureteral reflux (VUR), or renal dysplasia, all of which may adversely affect long-term renal function.

This broad clinical spectrum presents an important diagnostic and prognostic challenge. Antenatally detected hydronephrosis may represent a benign developmental variant or serve as an early marker of significant urinary tract obstruction. Distinguishing obstructive from non-obstructive causes of UTD is essential for prenatal counseling, delivery planning, and postnatal management, as this distinction directly influences surveillance strategies, the extent of postnatal investigation, and the timing of potential surgical intervention [8,9]. Clinically significant cases may also be associated with recurrent urinary tract infections, impaired renal growth or function, hypertension, and the eventual need for surgical correction [7,8,10,11].

Despite its high prevalence, there is no universally accepted definition of “clinically significant” FH, resulting in substantial variability in diagnostic thresholds, follow-up protocols, and reported outcomes across published studies.

The widespread adoption of routine prenatal ultrasonography has markedly improved the early detection of urinary tract abnormalities; however, it has also introduced important clinical dilemmas. These include determining which fetuses truly require intensified prenatal and postnatal monitoring, identifying those at risk of progressive renal damage, and avoiding unnecessary investigations or interventions in cases likely to resolve spontaneously [12]. Balancing the early identification of high-risk cases against the risks of overdiagnosis, parental anxiety, and excessive healthcare utilization remains a major challenge in contemporary fetal medicine.

In selected high-risk scenarios, particularly in fetuses with severe bilateral lower urinary tract obstruction (LUTO) complicated by oligohydramnios, prenatal interventions such as vesicoamniotic shunting have been explored as strategies to reduce perinatal mortality and mitigate pulmonary hypoplasia [13]. Although some studies have reported improved short-term survival in carefully selected cases, these invasive procedures are associated with significant procedural risks and variable long-term renal and respiratory outcomes, underscoring the need for cautious patient selection and long-term follow-up [14].

Historically, the assessment of FH relied primarily on isolated measurements of renal pelvic dilatation. Over the past two decades, this approach has evolved toward more comprehensive classification systems that integrate multiple anatomical and functional parameters. Among these, the UTD classification system represents a major advance, providing a standardized framework that incorporates renal pelvic measurements, calyceal involvement, renal parenchymal characteristics, ureteral and bladder findings, and amniotic fluid volume to improve risk stratification and prognostic accuracy [10].

Against this background, the present review aims to synthesize current evidence on FH, clarify contemporary terminology and risk-stratification approaches, and critically evaluate prenatal diagnosis, postnatal surveillance, and conservative and surgical management, with particular emphasis on clinically relevant long-term renal outcomes. Its added value lies in integrating fetal urinary tract development and prenatal imaging with standardized risk classification, postnatal diagnostic pathways, indications for intervention, and long-term outcomes within a single clinically oriented framework. Particular attention is given to ongoing areas of uncertainty, including the limitations of isolated renal pelvic measurements, variation in postnatal investigation protocols, and the challenge of identifying infants who require intervention while avoiding unnecessary surveillance in self-limiting cases.

Literature search and narrative synthesis

A targeted literature search was conducted in PubMed/MEDLINE and Google Scholar to identify publications addressing the embryology, prenatal diagnosis, classification, management, and long-term outcomes of FH and UTD. Articles published between 1970 and 2026 were considered using combinations of relevant search terms, including “fetal hydronephrosis,” “urinary tract dilation,” “UTD classification,” “prenatal ultrasound,” “lower urinary tract obstruction,” “postnatal management,” and “long-term renal outcomes.”

The selected literature included professional guidelines, consensus statements, meta-analyses, reviews, and clinically relevant observational studies. Foundational older studies were retained when relevant to embryology, diagnostic thresholds, or classification systems, and reference lists were screened for additional sources. Evidence was synthesized narratively, without meta-analysis or formal study-level risk-of-bias assessment. Quantitative findings were presented as estimates from individual studies or as ranges reported across heterogeneous publications and were not interpreted as pooled population-level effects.

## Review

Embryological and pathophysiological background

The fetal urinary tract develops from the intermediate mesoderm, with the definitive kidneys arising from the metanephros beginning in the fifth week of gestation. Reciprocal signaling between the ureteric bud and the metanephric mesenchyme drives branching of the collecting system and nephron formation. Disruption of this process may result in congenital abnormalities associated with obstruction, dysplasia, or impaired urinary drainage [15-19].

The metanephric kidneys begin producing urine at approximately the ninth week of gestation, and fetal urine becomes a principal determinant of amniotic fluid volume later in pregnancy [20,21]. Consequently, severe bilateral obstruction may impair renal development and contribute to oligohydramnios and pulmonary hypoplasia.

Hydronephrosis results from impaired urinary drainage and may occur at different anatomical levels. Persistent obstruction can increase intrarenal pressure, reduce renal perfusion, and lead to parenchymal thinning, tubular atrophy, interstitial fibrosis, and dysplasia. The timing, severity, and duration of obstruction are major determinants of renal outcome, with early and prolonged obstruction carrying the greatest risk of irreversible nephron loss [5,10,13,22].

However, not all prenatal UTD reflects true obstruction. Mild or transient dilation may result from physiological immaturity, transient vesicoureteral reflux, or delayed maturation of bladder function and may resolve spontaneously without adverse renal consequences [23]. This overlap between benign and pathological dilation highlights the importance of integrated prenatal assessment and longitudinal postnatal follow-up.

Criteria for evaluation and staging of FH

For many years, FH was characterized by substantial heterogeneity in diagnostic definitions and staging criteria, resulting in inconsistent reporting and variable postnatal management strategies. Early approaches to antenatal UTD were based largely on the assumption that dilation of the renal pelvis or ureter reflected abnormal renal development and that disease severity correlated with the degree of dilation. This conceptual framework, initially proposed in early descriptive studies of fetal urinary tract anomalies, prompted sustained efforts to identify objective and reproducible ultrasonographic markers capable of predicting postnatal outcomes and guiding clinical follow-up [24].

The reported prevalence of antenatally detected hydronephrosis ranges from approximately 1% to 4% of screened pregnancies, depending on gestational age at assessment and the diagnostic thresholds applied [25-27]. This variability underscores the need for standardized criteria that account for normal developmental changes in the fetal urinary tract and distinguish physiological dilation from clinically significant pathology.

One of the earliest structured approaches to prognostication was based on measurement of the fetal renal pelvic anteroposterior diameter (APD). Early observational studies demonstrated a clear association between increasing degrees of pelvic dilation and a greater likelihood of postnatal urinary tract pathology, as well as a higher probability of surgical intervention. These studies also emphasized the importance of gestational age, showing that identical APD values may carry different prognostic implications depending on whether they are identified in the second or third trimester [28]. Although APD measurement is simple, reproducible, and widely applicable, reliance on a single anatomical parameter was soon recognized as insufficient because it does not capture renal parenchymal integrity, calyceal involvement, or the dynamic nature of fetal urinary tract development.

Subsequent refinements incorporated additional ultrasonographic features to improve prognostic accuracy. Grignon et al. introduced a more comprehensive staging approach that combined APD measurements with the presence of calyceal dilation and renal parenchymal thinning. Their work demonstrated that the coexistence of marked pelvic dilation, calyceal involvement, and cortical thinning-particularly when the renal pelvic diameter reached or exceeded 15 mm-was strongly associated with adverse postnatal outcomes and frequently required surgical intervention to preserve renal function [29,30]. Calyceal dilation subsequently emerged as one of the most reliable indicators of clinically significant hydronephrosis, either as an isolated finding or in combination with pelvic measurements.

As evidence accumulated, it became increasingly apparent that fixed APD thresholds applied uniformly across gestational ages were inadequate predictors of postnatal outcome. Cohort studies and meta-analyses demonstrated that lower APD cutoffs were needed in the second trimester to maintain sensitivity, whereas higher thresholds were more appropriate in the third trimester to reduce overdiagnosis [29]. These findings led to the adoption of gestational age-adjusted APD criteria and further highlighted the limitations of single-parameter assessment strategies.

In response to these challenges, multidimensional classification systems were developed to integrate several anatomical and functional features of the fetal urinary tract. The Society for Fetal Urology (SFU) grading system represented a major advance by incorporating renal pelvic dilation, calyceal involvement, and renal parenchymal appearance into a structured framework. Although widely adopted, the SFU system has limitations in the prenatal setting because of its qualitative nature and interobserver variability. More recently, the UTD classification system was introduced to provide a standardized and comprehensive approach applicable to both prenatal and postnatal evaluation. This system integrates APD measurements with assessment of the calyces, renal parenchyma, ureters, bladder morphology, and amniotic fluid volume, thereby improving risk stratification and prognostic consistency [5,28,31].

Although these refinements have improved detection of associated anomalies such as vesicoureteral reflux, congenital obstructive megaureter, and ureterocele with upper UTD [32], they have also increased diagnostic sensitivity at the expense of specificity. Consequently, a growing number of neonates have undergone invasive and costly investigations, including voiding cystourethrography and radionuclide renography, despite ultimately demonstrating physiological or self-limiting dilation. This trend has been associated with increased parental anxiety and psychological burden, highlighting the need for balanced diagnostic strategies that avoid overtreatment while ensuring timely identification of clinically significant disease (Table 1) [9,33].

**Table 1:** Summary of the prenatal and postnatal urinary tract dilation (UTD) classification system. APD, anteroposterior diameter

Category	AP renal pelvic diameter	Calyceal dilation	Renal parenchyma	Ureter	Bladder	Amniotic fluid
UTD A1	Mild gestational-age-adjusted dilation	None or central	Normal	Normal	Normal	Normal
UTD A2-3	Increased APD and/or additional abnormalities	Peripheral may be present	Abnormal thickness, echogenicity, or cysts may be present	May be dilated	May be abnormal	May be decreased
UTD P1	Low-risk postnatal dilation	Central	Normal	Normal	Normal	Not applicable
UTD P2	Intermediate-risk postnatal dilation	Peripheral and/or ureteral involvement	Usually preserved	May be dilated	Usually normal	Not applicable
UTD P3	High-risk postnatal dilation	Variable	Abnormal parenchyma	May be dilated	May be abnormal	Not applicable

Despite substantial progress, further refinement of staging systems remains necessary. Contemporary evidence supports classification frameworks that extend beyond isolated pelvic measurements and incorporate additional sonographic and clinical variables, including fetal sex, gestational age and growth patterns, amniotic fluid volume, bilateral assessment of renal parenchymal thickness and echogenicity, cystic changes, the laterality and extent of UTD, bladder morphology before and after voiding, visualization of the posterior urethra when posterior urethral valves are suspected, associated extrarenal anomalies, and longitudinal trends on serial imaging. Integrating these parameters into multidimensional systems such as the UTD classification may help balance diagnostic sensitivity with clinical specificity, facilitate individualized risk stratification, optimize postnatal management, reduce unnecessary interventions, and improve parental counseling. Accurate staging also provides a foundation for distinguishing obstructive from non-obstructive causes of FH.

Although renal pelvic APD remains a simple and reproducible screening parameter, its prognostic value is limited when interpreted in isolation. Identical APD measurements may have different clinical implications depending on gestational age, calyceal involvement, renal parenchymal appearance, ureteral dilation, bladder abnormalities, and amniotic fluid volume. Multidimensional systems such as the UTD classification therefore provide a more clinically informative framework, although their performance remains dependent on operator expertise and consistent application (Table 2).

**Table 2:** Evolution of antenatal hydronephrosis assessment. APD, anteroposterior diameter; SFU, Society for Fetal Urology; UTD, urinary tract dilation

Era/System	Key parameter(s)	Gestational age	Strengths	Limitations
Early APD-based assessment	Renal pelvic APD alone	Not adjusted	Simple, reproducible	Poor prognostic accuracy; ignores renal parenchyma
Gestational age-adjusted APD	APD with trimester-specific thresholds	Yes	Improved sensitivity; better reflects fetal development	Still single-parameter-driven
SFU grading system	APD + calyceal dilation + parenchyma	Indirect	Widely adopted; structural assessment	Subjective; limited prenatal applicability
UTD classification system	APD + calyces + parenchyma + ureter + bladder	Explicit	Comprehensive, standardized, prenatal & postnatal	Requires expertise; more complex

Prenatal ultrasonographic evaluation

Prenatal ultrasonography is the cornerstone of fetal urinary tract assessment and remains the primary modality for the detection and longitudinal evaluation of FH. The first prenatal diagnosis of a urinary tract anomaly using ultrasonography was reported by Garrett in 1970, followed several years later by the description of FH [34,35]. Since then, the widespread integration of ultrasound into routine obstetric care has led to a substantial increase in antenatal detection rates of hydronephrosis and related urinary tract abnormalities. This increase reflects the noninvasive nature of ultrasonography, the absence of ionizing radiation, continuing technological advances, and growing operator experience.

Historical data underscore the considerable impact of routine prenatal ultrasound on detection rates. Retrospective analyses from Harvard Medical School demonstrated a marked increase in the diagnosis of antenatal hydronephrosis following the adoption of obstetric ultrasonography, with nearly identical numbers of cases identified over six years compared with the preceding three decades [36]. These findings suggest that improved detection reflects enhanced visualization rather than a true increase in disease incidence.

Despite these advances, the diagnostic performance of prenatal ultrasonography remains variable. Reported accuracy ranges from approximately 31% to 95% and is influenced by gestational age at examination, equipment resolution, operator expertise, and the absence of universally standardized criteria for defining pathological renal dilation [30,37-39]. False-positive diagnoses occur in an estimated 9%-22% of cases, most commonly because of transient physiological dilation observed between 12 and 16 weeks of gestation, which often resolves spontaneously [40]. Proposed mechanisms include temporary vesicoureteral reflux, immature bladder wall compliance, and transient alterations in fetal urine concentration.

To minimize misclassification, prenatal renal ultrasonography is ideally performed at more than one gestational time point and should include dynamic assessment during both full and empty bladder states. Serial examinations provide valuable information on the evolution of dilation, which is often more prognostically informative than a single measurement. In contrast, false-negative rates are more difficult to quantify because systematic postnatal screening of infants with normal prenatal findings is not routinely performed [41,42].

Professional societies have emphasized the importance of structured evaluation. The American Institute of Ultrasound in Medicine recommends targeted fetal renal assessment during both the second and third trimesters, including systematic evaluation of amniotic fluid volume, renal size and echogenicity, pelvicalyceal dilation, and bladder filling and emptying patterns [43]. Supporting this approach, large prospective population-based studies have shown that most urinary tract anomalies are detected before 33 weeks of gestation, highlighting the value of follow-up scans between 30 and 36 weeks, particularly in cases with evolving findings [44,45].

Recent International Society of Ultrasound in Obstetrics and Gynecology (ISUOG) practice guidelines similarly support the systematic evaluation of the fetal kidneys, urinary bladder, and amniotic fluid during routine mid-trimester examinations, with reassessment of fetal anatomy during third-trimester scanning when technically feasible. This is clinically relevant because some urinary tract abnormalities, including hydronephrosis, may become apparent or progress only later in gestation [46].

With modern high-resolution ultrasound systems, visualization of the fetal kidneys and bladder is possible as early as 14-15 weeks of gestation. However, assessment of renal parenchymal thickness, corticomedullary differentiation, and echotexture becomes more reliable after approximately 24 weeks, when perinephric fat improves image contrast [20,47-49]. During this period, cyclical fetal bladder filling and emptying, typically occurring every 20-30 minutes, can be observed and may provide indirect functional information. The ureters are rarely visualized prenatally unless distal obstruction or high-grade vesicoureteral reflux is present, and their detection often suggests more severe pathology. The laterality, severity, and temporal progression of hydronephrosis are closely related to the underlying etiology and gestational age at initial detection, reinforcing the importance of standardized grading systems and longitudinal assessment [42,49].

Advanced prenatal imaging and biomarkers

Although ultrasonography remains the cornerstone of prenatal urinary tract evaluation, fetal magnetic resonance imaging (MRI) has emerged as a valuable second-line modality in selected clinical scenarios. Its use is primarily reserved for cases complicated by oligohydramnios, maternal obesity, suboptimal acoustic windows, or equivocal sonographic findings. In such settings, fetal MRI provides superior soft-tissue contrast and multiplanar visualization, allowing improved assessment of renal parenchymal integrity, bladder wall morphology, and associated extrarenal anomalies, including those involving the spine and gastrointestinal tract [50-52].

Fetal MRI is not recommended as a routine screening tool. Its role is complementary to, rather than a substitute for, ultrasonography and should be limited to cases in which additional anatomical clarification is expected to influence prenatal counseling or postnatal management. Recent advances in imaging technology have further refined its role in characterizing complex genitourinary anomalies and supporting perinatal decision-making [51,53]. However, current evidence does not support the routine use of fetal MRI for isolated UTD or its use as an independent predictor of postnatal renal function. Cost, limited availability, and the need for specialized expertise further restrict its widespread application, particularly in resource-limited settings.

In parallel, increasing attention has been directed toward biochemical markers derived from amniotic fluid, fetal urine, and postnatal urine, to provide functional information beyond structural assessment. Proteomic approaches have identified several candidate biomarkers associated with renal developmental disease and lower urinary tract obstruction, including peptides, β2-microglobulin, cystatin C, sodium, chloride, osmolarity, albumin, and urinary neutrophil gelatinase-associated lipocalin [52-55].

Although several fetal urinary, amniotic fluid, and postnatal urinary biomarkers have shown associations with obstruction or the need for surgery, most available studies are small, single-center, and methodologically heterogeneous. Variability in sampling techniques, gestational age-specific reference ranges, and outcome definitions has limited their clinical applicability. At present, these biomarkers remain investigational, cannot replace standardized imaging or functional assessment, and should not independently guide prenatal counseling or surgical intervention.

Future developments in proteomic and metabolomic diagnostics may allow more refined risk stratification. However, the integration of biomarkers into routine clinical practice will require validation in large, prospective, multicenter studies using standardized sampling methods and clinically meaningful outcome measures.

Differential diagnosis: Obstructive vs. non-obstructive causes

A fundamental objective of prenatal evaluation is to distinguish obstructive from non-obstructive causes of UTD, as this distinction directly influences prognosis, surveillance intensity, and postnatal management. Antenatally detected hydronephrosis encompasses a heterogeneous group of conditions, ranging from transient physiological variants to clinically significant obstructive uropathies with potential long-term renal consequences.

Obstructive hydronephrosis most commonly results from ureteropelvic junction obstruction, posterior urethral valves, or obstruction at the ureterovesical junction. These conditions impair urinary drainage and may lead to progressive renal pelvic dilation, calyceal involvement, cortical thinning, and deterioration of renal function if left untreated. Prenatal ultrasonographic features that raise suspicion for obstruction include a progressive increase in anteroposterior pelvic diameter on serial examinations, calyceal dilation, reduced renal parenchymal thickness, bilateral involvement, abnormal bladder morphology, and oligohydramnios. When several of these findings coexist, the likelihood of clinically significant postnatal pathology increases, warranting closer prenatal surveillance and structured postnatal evaluation.

In contrast, non-obstructive causes of UTD include transient physiological hydronephrosis, vesicoureteral reflux, and delayed or immature urinary drainage. Physiological hydronephrosis is typically mild, often unilateral, and self-limiting, particularly in male fetuses and during late gestation. Favorable sonographic features include preserved renal parenchymal thickness, normal cortical echogenicity, absence of ureteral dilation, normal bladder wall thickness, and adequate amniotic fluid volume. However, because early sonographic appearances may overlap, careful longitudinal assessment remains essential to distinguish benign variants from evolving obstructive pathology (Table 3).

**Table 3:** Differential diagnosis of fetal urinary tract dilation: prenatal features and recommended postnatal evaluation. UPJ, ureteropelvic junction; VUR, vesicoureteral reflux; UVJ, ureterovesical junction; US, ultrasound; MAG3, technetium-99m mercaptoacetyltriglycine renography; VCUG, voiding cystourethrography

Condition	Typical prenatal clues	Important postnatal test
UPJ obstruction	Pelvic/calyceal dilation, usually no hydroureter	MAG3
VUR	May have variable or intermittent dilation	VCUG
Posterior urethral valves	Bilateral dilation, thick bladder, keyhole sign, oligohydramnios	Urgent VCUG
UVJ obstruction/megaureter	Hydroureteronephrosis	US ± MAG3/VCUG
Transient dilation	Mild, stable or improving, preserved parenchyma	Serial US

Prenatal management and fetal intervention

Prenatal management is guided by the severity and laterality of UTD, the suspected underlying etiology, renal parenchymal appearance, bladder and urethral findings, and amniotic fluid volume. Most cases of isolated mild or moderate UTD do not require prenatal intervention and are managed with serial ultrasonographic surveillance to monitor progression, renal parenchymal integrity, bladder emptying, and amniotic fluid volume [5,10,27,56].

Multidisciplinary consultation is appropriate when severe bilateral dilation, abnormal bladder morphology, oligohydramnios, or suspected lower urinary tract obstruction is identified [53,56].

Fetal intervention is reserved for highly selected cases of severe lower urinary tract obstruction associated with oligohydramnios and a substantial risk of pulmonary hypoplasia and irreversible renal dysplasia. Vesicoamniotic shunting is the most established intervention and may improve perinatal survival in carefully selected fetuses; however, evidence regarding its long-term renal benefit remains uncertain [13,53]. The procedure is associated with complications including shunt displacement or obstruction, infection, rupture of membranes, preterm labor, and maternal morbidity. Consequently, fetal intervention should be considered only after detailed multidisciplinary assessment and parental counseling regarding the potential survival benefit, uncertain renal outcome, procedural risks, and expected long-term prognosis [13,53].

Postnatal evaluation and management

Postnatal evaluation should be risk-based rather than uniform. Renal and bladder ultrasonography is the initial imaging investigation. In clinically stable infants, it is generally performed after the first 48 hours of life because ultrasonography undertaken during the immediate neonatal period may underestimate UTD owing to low urine output and relative neonatal dehydration [56,57].

Earlier assessment is warranted in neonates with suspected lower urinary tract obstruction, severe bilateral dilation, oligohydramnios, abnormal bladder or urethral findings, or other features indicating high-risk disease. Subsequent imaging and follow-up should be guided by the postnatal UTD risk category, persistence or progression of dilation, renal parenchymal appearance, ureteral and bladder abnormalities, and the infant’s clinical course [5,27,56,57].

Routine voiding cystourethrography or radionuclide renography is not warranted in every infant with antenatally detected UTD. Voiding cystourethrography is generally reserved for cases with ureteral dilation, abnormal bladder or urethral findings, recurrent or febrile urinary tract infection, or suspicion of vesicoureteral reflux or bladder outlet obstruction. Technetium-99m MAG3 renography is more appropriate when persistent high-grade dilation, impaired drainage, progressive parenchymal thinning, or reduced differential renal function raises concern for clinically significant obstruction [56-58].

Most infants with isolated low-risk UTD demonstrate spontaneous improvement or resolution and can be managed conservatively with serial ultrasonography and clinical follow-up [11,27,57,59].

The role of continuous antibiotic prophylaxis remains controversial. It is generally not recommended for isolated low-risk UTD, in which the absolute risk of febrile urinary tract infection is low. Its use may be considered selectively in infants with high-grade dilation, ureteral involvement, suspected vesicoureteral reflux, or recurrent urinary tract infection, although the available evidence remains heterogeneous and practice varies among centers [55-59].

Surgical intervention, most commonly pyeloplasty for ureteropelvic junction obstruction, is considered when serial evaluation demonstrates progressive dilation, deterioration of renal parenchymal appearance, impaired drainage, declining differential renal function, recurrent febrile urinary tract infections, or symptoms attributable to obstruction. No single APD or renographic parameter should be used as an isolated indication for surgery [33,55,58].

Although differential renal function below 40% is commonly regarded as an important indicator, it should be interpreted within the broader clinical and imaging context. Surgical decision-making should integrate serial anatomical findings, renal parenchymal integrity, drainage patterns, functional assessment, symptoms, and the overall clinical course [33,55,58].

This risk-stratified approach aims to preserve renal function while avoiding unnecessary invasive investigations and interventions in self-limiting cases.

Current controversies in perinatal UTD management

Despite increasing standardization, several aspects of perinatal UTD management remain controversial because many recommendations are based primarily on observational studies, expert consensus, and heterogeneous institutional protocols rather than high-quality comparative trials.

The optimal timing of the first postnatal renal and bladder ultrasound depends on clinical risk. Imaging performed during the first 24-48 hours of life may underestimate the degree of dilation because of low neonatal urine output and relative dehydration. Clinically stable infants with low- or intermediate-risk findings are therefore generally evaluated after 48 hours, whereas earlier imaging is appropriate in neonates with suspected lower urinary tract obstruction, severe bilateral dilation, oligohydramnios, or abnormal bladder findings [56,57].

The indications for voiding cystourethrography also remain debated. The severity of prenatal or postnatal dilation alone does not reliably predict vesicoureteral reflux, and routine VCUG in all infants may expose many low-risk children to unnecessary catheterization and radiation. Contemporary risk-based approaches therefore reserve VCUG primarily for infants with ureteral dilation, abnormal bladder or urethral findings, suspected lower urinary tract obstruction, or febrile or recurrent urinary tract infection [56,57].

Continuous antibiotic prophylaxis represents another area of uncertainty. Available evidence does not support its routine use in isolated low-risk UTD, in which the absolute risk of febrile urinary tract infection is generally low. Prophylaxis may be considered selectively in infants with high-grade dilation, hydroureteronephrosis, suspected vesicoureteral reflux, or other features associated with an increased risk of infection. However, variation in study populations, diagnostic definitions, and follow-up protocols continues to limit firm recommendations [56,58].

**Figure 1:**
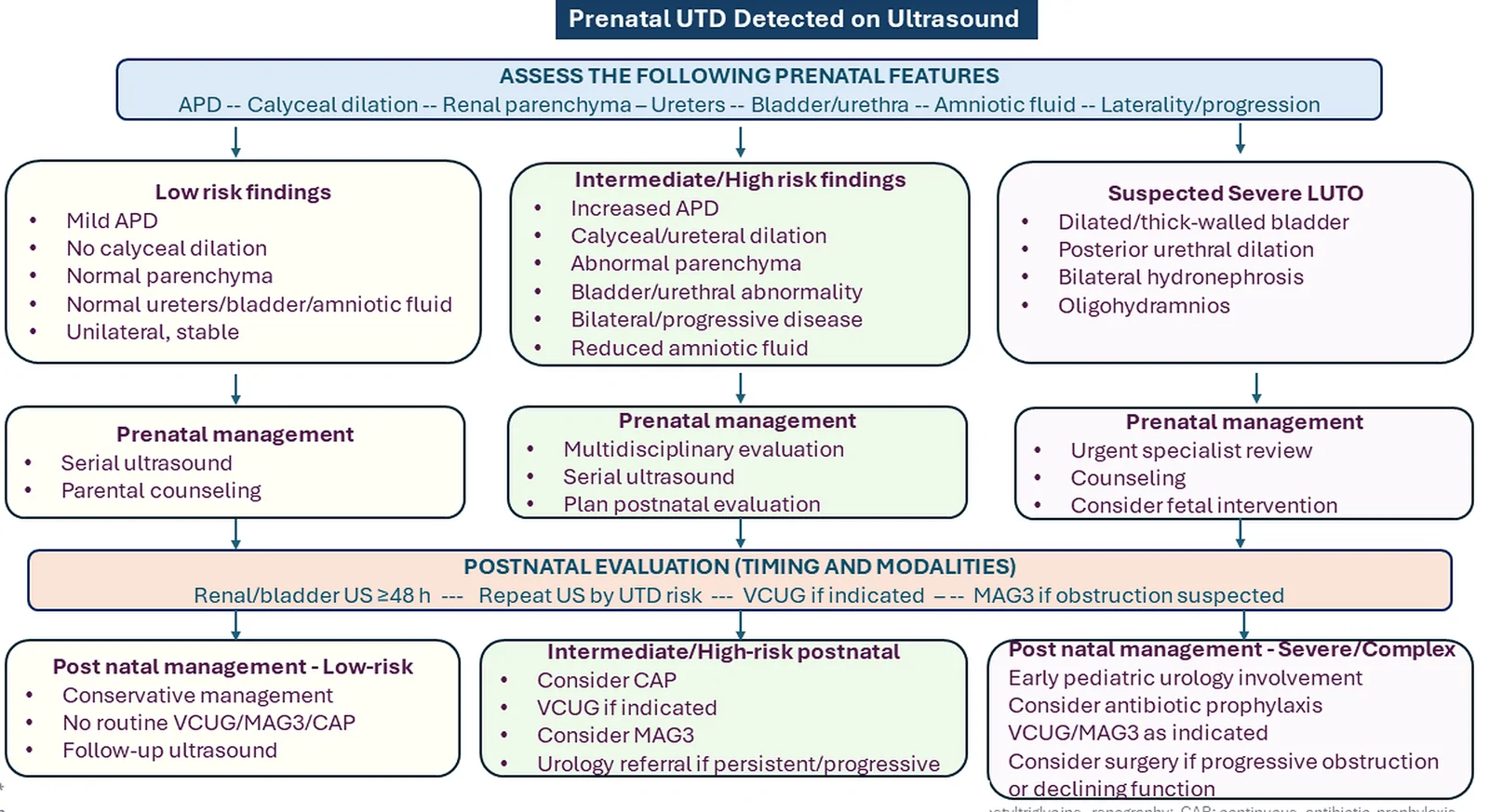
Risk-based evaluation and management of perinatal urinary tract dilation (UTD). Proposed clinical framework integrating prenatal UTD features with postnatal imaging and management. Decisions should be individualized according to the UTD risk category, associated abnormalities, renal parenchymal integrity, functional findings, and local multidisciplinary protocols. Author-created educational summary based on current evidence and guideline recommendations. CAP should be individualized according to UTD risk and clinical findings. Image generated by the author using Microsoft PowerPoint (Microsoft Corporation, Redmond, WA). APD, anteroposterior diameter; LUTO, lower urinary tract obstruction; VCUG, voiding cystourethrography; MAG3, technetium-99m mercaptoacetyltriglycine renography; CAP, continuous antibiotic prophylaxis

Finally, no single ultrasonographic or renographic threshold should independently determine the need for surgery. Although declining differential renal function, impaired drainage, progressive dilation, parenchymal thinning, recurrent infection, and symptoms are commonly used indicators, the relative importance of each factor varies across centers. Surgical decision-making should therefore integrate serial anatomical findings, functional assessment, and the clinical course rather than rely exclusively on an isolated APD value or a single differential renal function threshold (Figure 1) [33,55,58].

Long-term prognosis and outcomes

Most cases of antenatally detected hydronephrosis are associated with favorable long-term outcomes. However, high-grade dilation, bilateral involvement, and coexisting renal dysplasia are associated with an increased risk of chronic kidney disease. Accumulating evidence indicates that early detection, structured postnatal follow-up, and timely intervention are important for preserving renal function. Established predictors of adverse outcome include increased renal parenchymal echogenicity, cortical thinning, oligohydramnios, and differential renal function below 40%.

Recent studies have reinforced the prognostic value of combining anatomical and functional parameters to predict the need for surgical intervention. In a 2025 study of children with antenatally detected hydronephrosis and histopathologically confirmed ureteropelvic junction obstruction, anteroposterior pelvic diameter, renal parenchymal thickness, and diuretic renography parameters - specifically half-time and time to peak activity - were identified as independent predictors of subsequent pyeloplasty [33]. These findings support the clinical value of integrating sonographic measurements with functional renographic indices when considering surgical timing.

Longitudinal cohort data further support a favorable prognosis in most cases of isolated antenatal hydronephrosis. In a 2024 cohort study involving more than 200 patients and over 250 affected renal units, spontaneous resolution occurred in more than 90% of mild cases and over 80% of moderate cases, whereas resolution was observed in fewer than 40% of severe cases. Surgical intervention was required in only a small proportion of patients, and long-term renal outcomes were favorable, with rare instances of reduced glomerular filtration rate and no reported cases of hypertension or proteinuria during follow-up [59].

Comparative analyses have also examined the effects of evolving management protocols on clinical outcomes and resource use. A decade-long experience showed that implementation of more conservative, updated antenatal hydronephrosis guidelines resulted in fewer follow-up imaging studies, reduced radiation exposure, and lower surgical rates, without compromising detection of clinically significant pathology such as vesicoureteral reflux or obstructive uropathy [60]. These findings highlight the importance of protocol-based surveillance strategies that balance diagnostic sensitivity with clinical specificity.

The timing of prenatal detection may also influence postnatal management. Large population-based studies have shown that a substantial proportion of fetal urinary tract abnormalities are first identified during late third-trimester scans, emphasizing the value of follow-up ultrasonography in the final weeks of pregnancy for perinatal planning and postnatal evaluation [61].

Finally, emerging evidence suggests that combining sonographic parameters with urinary biomarkers may further improve prognostic accuracy. Studies integrating ultrasound measurements with biomarkers such as β2-microglobulin, albumin, and urinary neutrophil gelatinase-associated lipocalin have shown improved prediction of the need for surgery compared with anatomical measurements alone. In particular, ratios incorporating anteroposterior pelvic diameter and parenchymal thickness have demonstrated high predictive value and specificity for surgical intervention [55]. Although these findings are promising, broader validation is required before routine clinical implementation.

Available long-term data are reassuring for most children with mild isolated UTD. However, the evidence is less robust for high-grade, bilateral, or dysplastic cases. Many studies are retrospective, use differing definitions, and do not extend into adolescence or adulthood. Consequently, the true lifetime risks of hypertension, proteinuria, reduced glomerular filtration rate, and chronic kidney disease remain incompletely defined.

Limitations of current evidence

Despite substantial progress in the prenatal evaluation and management of FH, several limitations continue to constrain the interpretation and generalizability of available evidence. A major challenge arises from the considerable heterogeneity among published studies, including differences in diagnostic thresholds, gestational age at assessment, imaging protocols, follow-up duration, and outcome definitions. These variations complicate direct comparison across studies and limit the establishment of universally applicable prognostic cutoffs.

Many of the available data are derived from retrospective or single-center observational studies, which are inherently susceptible to selection bias and incomplete long-term follow-up. In particular, long-term renal outcomes extending into adolescence and adulthood, including the true incidence of chronic kidney disease, hypertension, and subtle functional impairment, remain insufficiently characterized for most cohorts. In addition, differences in postnatal management strategies across institutions may influence reported outcomes, further limiting comparability.

Although multidimensional classification systems such as the UTD framework have improved risk stratification, interobserver variability and dependence on operator expertise remain relevant limitations, particularly in low-resource settings. Similarly, while emerging urinary and amniotic fluid biomarkers show promise, their clinical utility is restricted by inconsistent methodologies, lack of standardized reference ranges, and variable predictive performance across gestational ages and disease severities. Consequently, most biomarkers remain investigational and should be interpreted cautiously within the broader clinical context.

As a narrative review, this article did not employ systematic study screening, predefined eligibility criteria, formal study-level risk-of-bias assessment, or quantitative evidence synthesis. Source selection and interpretation may therefore be subject to narrative selection bias, and the numerical estimates presented should be interpreted in the context of heterogeneous study populations, definitions, and follow-up periods.

Future directions and clinical implications

Future research in FH should focus on harmonizing diagnostic criteria and follow-up protocols to improve consistency across studies and clinical practice. Large, prospective, multicenter studies using standardized imaging protocols and outcome measures are needed to refine risk-stratification models and clarify the long-term renal consequences of antenatally detected UTD.

The integration of anatomical imaging with functional assessment represents an important direction for advancing individualized care. Refinement of imaging-based metrics, combined with validated functional parameters from postnatal renography, may enable more accurate prediction of disease progression and better determination of the optimal timing of intervention. In parallel, continued investigation of urinary and molecular biomarkers may complement imaging findings, particularly in identifying fetuses and infants at greatest risk of irreversible renal damage.

From a clinical perspective, protocol-based surveillance strategies grounded in multidimensional classification systems may reduce unnecessary investigations and interventions while ensuring timely identification of clinically significant pathology. Such approaches may improve parental counseling, optimize resource utilization, and promote more consistent, evidence-based decision-making across centers. As diagnostic tools continue to evolve, future frameworks should aim to balance sensitivity and specificity while remaining practical and broadly applicable.

## Conclusions

FH encompasses a broad and heterogeneous clinical spectrum, ranging from benign physiological variants to severe obstructive uropathies that may require prenatal or early postnatal intervention. Advances in prenatal imaging, standardized risk-stratification systems, and emerging biomarker research have improved diagnostic accuracy and prognostic assessment.

Optimal management relies on a multidisciplinary, longitudinal approach that integrates anatomical, functional, and clinical parameters across the prenatal and postnatal periods. Such strategies enable timely identification of high-risk cases while minimizing unnecessary investigations and interventions in self-limiting conditions.

Further refinement of classification frameworks, together with the incorporation of functional imaging metrics and novel molecular and genetic markers, may enhance individualized care, improve long-term renal outcomes, and support evidence-based counseling for affected families.
